# On the Application of Different Event-Based Sampling Strategies to the Control of a Simple Industrial Process

**DOI:** 10.3390/s90906795

**Published:** 2009-08-27

**Authors:** José Sánchez, Miguel Ángel Guarnes, Sebastián Dormido

**Affiliations:** 1 Department of Computer Science and Automatic Control, UNED, C/ Juan del Rosal, 16, 28040 Madrid, Spain; E-Mail: sdormido@dia.uned.es (S.D.); 2 National University of San Luis, San Luis, Argentina; E-Mail: mguarnes@unsl.edu.ar (M.A.G.)

**Keywords:** send-on-delta, event-based control, PI controller, 07.05.Dz, 87.19.lr, 02.30.Yy

## Abstract

This paper is an experimental study of the utilization of different event-based strategies for the automatic control of a simple but very representative industrial process: the level control of a tank. In an event-based control approach it is the triggering of a specific event, and not the time, that instructs the sensor to send the current state of the process to the controller, and the controller to compute a new control action and send it to the actuator. In the document, five control strategies based on different event-based sampling techniques are described, compared, and contrasted with a classical time-based control approach and a hybrid one. The common denominator in the time, the hybrid, and the event-based control approaches is the controller: a proportional-integral algorithm with adaptations depending on the selected control approach. To compare and contrast each one of the hybrid and the pure event-based control algorithms with the time-based counterpart, the two tasks that a control strategy must achieve (set-point following and disturbance rejection) are independently analyzed. The experimental study provides new proof concerning the ability of event-based control strategies to minimize the data exchange among the control agents (sensors, controllers, actuators) when an error-free control of the process is not a hard requirement.

## 1. Introduction

In recent times research on automatic control techniques based on asynchronous sampling has increased steadily [1–12], to the point where it cannot be considered a new research topic [13–16]. The reason of such interest in these techniques is a direct consequence of the impact that wireless sensor networks (WSN) and networked control systems (NCS) offer to the control engineering community. Firstly, wireless technologies applied to sensors and controllers allow the deployment of distributed control systems in a very flexible way and in places where the installation or augmentation of a control system based on traditional hard-wired components could be impossible or very expensive [17]; secondly, the development of networked control systems based on Internet and off-the-self components is receiving a great attention from the industrial and academic world since this type of NCS offer interesting features as, for example, flexibility, lower cost, ease of extension, etc.

However, the utilization of classical time-based or time-triggered paradigms in these distributed control systems imposes system architectural constraints that make difficult to stick them to the time-triggered paradigm. This is specially the case when control loops are closed over shared networks, like the Internet, and they must cope with a very important problem: the existence of delays in the network transmissions that produce lack of synchronization among the main control agents (and all the computer control theory is based on such a rigid assumption). In this situation, event-based approaches represent a promising research line to develop new control strategies where the exchange of information among control agents is produced by the triggering of specific events and not by the passing of time.

Another reason why event-based control is interesting is that it closer in nature to the way a human behaves as a controller. The final reason to research in event-based control is computing and communication resource utilization, that is, the reduction of the data exchange between sensors, controllers, and actuators. This reduction of information is equivalent to extend the lifetime of battery-powered wireless sensors, to reduce the computational load in embedded devices, or to reduce the network bandwidth.

Why is it then that time-triggered control still dominates? A major reason is the great difficulty involved with developing a system theory for event based control systems. Until now, most of the research lines in event-based control have tried to adapt time-based control approaches to the event-based paradigm, producing systems where time-based and event-based elements are all living together in the control loop [18]. Other developments have tried to devise pure event-based control approaches with a total lack of synchronism or sharing of clock signals among sensors, controllers, and actuators [19,20]; in this research line the control agents are always activated by specific events and it is where most difficulties emerge to produce theoretical developments to back the experimental results. The work presented in this paper corresponds to the second category: an experimental study of pure event-based approaches.

As it was said at the beginning, until now the majority of the published work in automatic control considers time-based control systems as the only paradigm to implement automatic control systems. However, when taking a quick look at human behavior, it is clear that the triggering of events is the strategy we use to apply feedback control in many facets of everyday life. For example, in a traffic jam drivers hold the safety distance among cars by braking or speeding up, but drivers do not have precision clocks to signal when they have to observe the distance with the car in front of them; they are observing the back of the next car and when a driver subjectively considers that the safety distance is short enough s/he sends a new control action to the car - to brake -; and if the distance is long enough, then the control action is to speed-up. Another similar event-based control strategy is used every morning when we are trying to regulate by hand the water temperature when we take a shower.

It is important to notice that drivers take samples in a continuous way and only when the distance crosses a threshold (that is, an event) the control action is calculated and sent to the car. Since the current data acquisition systems are time-driven, the human event-based sampling must be simulated by a technique known as “upward even-driven architecture” [21]. It consists of sampling the signal using a periodic scheme and to evaluate if every sample fulfils the condition to consider that an event is happening and so to trigger an action.

In event-based control systems is the occurrence of an asynchronous event that pushes forward to the main agents involved in the control scheme (sensors, controllers, actuators) to perform an action. In this work, we consider that an event happens when the value of some parameter (output, state, error, integrated absolute error, control action, etc.) changes, deviates, or exceeds a threshold. As a result of it, the agent detecting the event is who produces the action. To clarify the work developed in the document, Table 1 shows the events and actions associated to the control agents that we have considered in the pure event-based control strategies.

In Table 1, when the agent is the sensor, *fulfill error-based criterion* means that at some instant *t* a logical error-based expression becomes true (for example, the error or the absolute integrated error exceeds a certain threshold), and, as consequence of that, the process output *y*(*t*) is sent to the controller. It must be noticed that, in some situations, the sensor owns a synchronous event, that is, a time-out, to force the sending of a sample to the controller. The reason of that is to introduce a safety element and so to push the controller to send a new control action avoiding the sticking. This phenomenon happens when the error derivative trends to zero, and the control loop achieves a temporary equilibrium where sensor and controller do not exchange information, leaving the system in a state where error exists [22]. Due to the inclusion of this time-based event, if the error-based condition of the sensor is always fulfilled, we would have the well-known time-driven approach.

Following Table 1, in the actuator, an event is the arrival of a new control value, and the action is the application of it to the process. In our test-bed, the actuator owns a ZOH (Zero-Order Hold), so the current control action is maintained till the arrival of a new one.

Since a controller has inputs and outputs, we have considered input- and output-side events. The input-side ones are the arrival of a new *y* (as consequence of the triggering of some of the sensor-side events) and the introduction of a new reference *y*_ref_. Both cases force the calculation of a new control action *u*, with independence of the algorithm used in the controller. The *u-based criterion* of the output-side consists of just sending the new control action if it is different enough regarding the previous control action.

This paper is organized as follows. Since this work is an experimental study where pure event-based control approaches are compared with a time-based and a hybrid one, Section 2 describes in detail the architecture of the three groups of approaches, paying special attention to the five event-based strategies; also a short description of the software architecture used will be provided. Section 3 describes the experimental set-up and the performance criteria used to make the comparatives among the approaches. The test-bed used for conducting the experiments is a Quanser’s double tank, where level control of the upper tank is achieved. Tables, figures, and comments of the results corresponding to the set-point following and the disturbance rejection tasks are presented in Section 4. In the end, some conclusions and considerations about further work are given.

## 2. Architecture of the Control Approaches

As it was said before, three very different groups of control approaches are used in the experimental study: time-based, event-based, and hybrid. Table 2 summarizes the nature of the agents of the three groups.

Regardless how the control agents are driven (time, events, or both), it is important to indicate that in our experimental framework the relationship among the three control agents is fully asynchronous, that is, there is no sharing of clock signals between then, just control information is exchanged (control action and process output). That means that, regardless the approach, every control agent is running at its own pace: in the event-based the pace is set up by the triggering of consecutive events; in the time-based the pace is fixed by the specific timer of every agent.

Also in every approach the calculation of the control action is made up by a PI with anti-windup and limitation of the control action. A detailed description of the PI algorithm and its pseudocode can be found in [23].

The difference of the PI algorithm in each control approach is the sampling time *h*_controller_ used to compute the integral contribution of the controller. In the time-based approach, *h*_controller_ is fixed and does not change; in the event-based strategies *h*_controller_ is the time elapsed between two consecutive events, that is, between two consecutive arrivals to the controller of the process output; in the hybrid one, there is a fixed nominal sampling time *h*_controller_ but the sampling time used to compute the integral action, *h*_without_, depends on the occurrence of an event and it is always a multiple of *h*_controller_.

**Figure 1.**
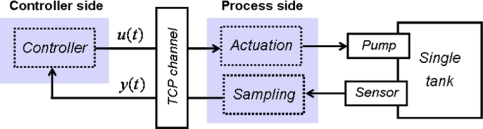
Diagram illustrating the architecture used in the development of the experimental framework. The controller side is composed by one process: the PI controller. The process side contents two processes: a loop to receive the control actions and apply them to the pump by a ZOH; and a second one that reads the level of the single tank and sends these readings to the controller.

Also, the separation between sensor and controller in the time-based because the existence of *h*_sensor_ and *h*_controller_, and also in the other approaches as consequence of their event-based nature, lets us program the experimental framework in a decoupled way. That means that, regardless the control approach, the software processes associated to the sensor, the controller, and the actuator are independent and they can be arranged into controller side and process side (Figure 1). The reason to separate and distinguish between controller and process side is two-fold:

1. In the software implementation of our experimental framework, the sampling and control tasks are independent applications running in the same computer but exchanging data by local TCP sockets. That will let us move the controller to a remote computer in further research, placing Internet between the sensor and the controller and allowing us to test the event-based strategies in presence of transmission delays, and
2. The dynamics of the single tank is not very high: a first order system with a time constant of 14 s. That involves that the results are not be very dissimilar from a full-synchronous time-based approach taking into account that the sampling and control tasks are running with values of *h*_controller_ and *h*_sensor_ equal to 0.1 s. in all the experiments.

It is usual in the event-based literature that some boxes in Figure 1 receive other names [5,6,10]. So, “sampling” is known either as “control event generator” when it is connected to the controller or “signal event generator” when it is connected to an “event-based observer”. The “controller” is known as “control signal generator”. In our case, the “control signal generator” is the PI controller plus the ZOH; in other cases, it can be an impulse generator, a generalized hold, a MPC plus a ZOH, etc. In Figure 1, the only continuous signals are the actuator-to-process and the process-to-sensor. The nature of the other signals depends on the nature of the control approach chosen.

### 2.1. The Time-Based Approach

The time-driven approach is similar to any other feedback control loop using a classical PI controller but there is no sharing of clock signal among the involved agents. So, excluding the actuator, the other two agents own their clock that able them to operate at different frequencies. This time-based strategy could be more accurately labeled as an *asynchronous time-based control approach*.

**Figure 2.**
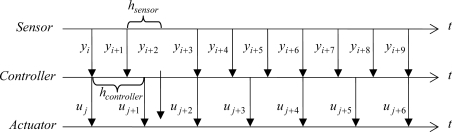
Example of exchange of control information between sensor, controller, and actuator using the asynchronous time-based approach where *h*_sensor_ ≠ *h*_controller_. It is considered that there is no delay in the control loop and the computation time is negligible.

In the time-driven approach, the control parameters are *Kc*, *Ti*, *h*_sensor_, and *h*_controller_. Figure 2 presents a scheme of this asynchronous time-based approach where sensor and controller are driven by time but with different *h*_sensor_ and *h*_controller_, and the actuator just works when a new *u* is received.

### 2.2. The Event-Based Approach

**Figure 3.**
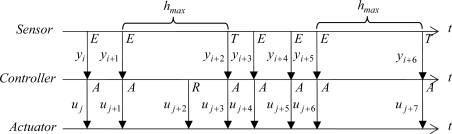
Exchange of information between sensor, controller, and actuator in the event-based approach. The *E* represents when the error-based condition of the sensor becomes true and the *T* represents when the sending is forced by a time-out. An *A* represents a new arrival, and *R* a change of the set-point value.

In pure event-based strategies, the three control agents are driven by asynchronous events with some exceptions. Figure 3 shows an example of the exchange of data among the three control agents. In this approach, the sensor can send a sample to the controller as consequence of the firing of two types of events: asynchronous events and time-triggered ones. In the first category, the asynchronous events are triggered when some *error-based condition* becomes true. The events in the second category are just fired when the time lapsed from the last event triggering is greater than a certain time *h_max_*. However, depending on the error-based condition selected, the inclusion of the time-triggered condition can result unnecessary since the formulation of the error-based condition avoids by itself the appearance of the sticking phenomenon, which happens when *ė*(*t*) is small and tends to zero.

In the controller side, the calculation of the control action is done in two situations: the arrival of a new sample and the modification of the set-point value. In both cases, a new control action is calculated and sent to the actuator. Since the actuator is event-based, the control action is always applied to the process.

**Figure 4.**
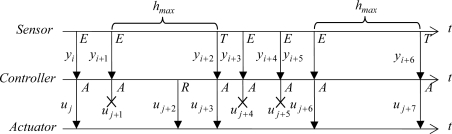
Exchange of information of the event-based approach including a simple send-on-delta strategy in the controller. The *u*_*j*+1_ is calculated but not transmitted since |*u*_*j*+1_ − *u_j_*| < Δ*u*. The same situation happens with *u*_*j*+4_ and *u*_*j*+5_.

Regardless of the control approach, the controller can include a *u-based criterion* to reduce the number of controller-actuator transmissions. Figure 4 presents an example based on Figure 3 where the control action, *u*_current_, is transmitted only if it is Δ*u* greater than the last control action sent to the actuator, *u*_last_. It is important to point out that the inclusion of a send-on-delta strategy in the controller is completely independent of the control algorithm and of the sampling strategy used in the sensor.

According to the error-based condition used in the sensor, different event-based strategies are obtained (see Table 3). First and second conditions of Table 3 do not need a detailed explanation since both are simple well-known deadband sampling strategies. Further details on these methods can be found in [24–27]. The LP method, originally described in [28], consists of starting the calculation of future error values after an event takes place. To do that a first order predictor:

$$\hat{e}\left(t_{k}\right)=f\left(\hat{e}\left(t_{k-1}\right),\;\hat{e}\left(t_{k-2}\right)\right)$$

is used to estimate the evolution of the signal error from last time a sample was sent to the controller. When the difference between the value of the current error and its prediction for the current time is greater than Δ_event_, the condition becomes true and the current plant output *y*(*t_k_*) is transmitted to the controller.

The ILP is a new proposal of criterion based on the previous LP. In this case, the sample is taken and sent when the area between the error signal and its prediction is greater than Δ_event_. The EN criterion [28] sends a sample of the output plant when the energy of the error signal from last sending exceeds a certain threshold.

As it was said before, depending on the formulation of the error-based condition, an additional time-triggered expression must be added to the error-based condition to force a sending when a time-out expires. The main reason to do that is the avoidance of the sticking. This new mixed condition would be similar to:

$$\text{error\_based condition IS true OR}\;h_{\mathrm{without}}\geq h_{\max}$$

where *h*_without_ represents the elapsed time from the last sending of a sample to the controller. The presence of sticking specially happens when the sampling methods are not based on integration, as for example, the LC and LP criteria. In both methods, there are situations where the sensor does not sent information to the controller in spite of the existence of error because *ė*(*t*) equals zero; such situation produces that the value |*e*(*t*) − *e*(*t_k_*)| or |*ê*(*t*) − *e*(*t*)| remains below the threshold Δ_event_ for long time. However, the sticking is avoided in the criteria where integration is done (ILC, ILP, and EN) since the error-based condition becomes true even though *ė*(*t*) is 0. As well as in the hybrid approach, *h*_max_ is equal to the settling time of the open-loop process.

### 2.3. The Hybrid Approach

This control approach is based on the first event-based PI controller described in the literature [18]. As in the time-based approach, the sensor is time-driven with a period *h*_sensor_ but the controller uses a mixed strategy. Every *h*_controller_ the controller evaluates the error-based condition:

(1) $$\left|e(t)-e\left(t_{k}\right)\right|\geq \Delta_{\mathrm{event}}$$

where *e*(*t*) is the current value of the error and *e*(*t_k_*) is the value of the error last time the control action was calculated. When expression (1) becomes true the control action is calculated by the PI and sent to the actuator; however, if the controller exceeds a time *h*_max_ without calculating a new control action (*h*_without_), a calculation is forced by safety reasons. So, the controller is driven by error-based events but, in some situations, also by time. The complete error-based condition becomes:

(2) $$\left|e(t)-e\left(t_{k}\right)\right|\geq \Delta_{\mathrm{event}}\;\text{OR}\;h_{\mathrm{without}}\geq h_{\max}$$

**Figure 5.**
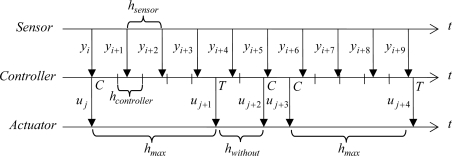
Exchange of information between sensor, controller, and actuator in the hybrid approach where *h_max_* = 5 × *h_controller_*. The *C* represents when the control action is calculated because the error-based condition becomes true, and the *T* represents when the controller acts by time because *h*_without_ ≥ *h*_max_.

In our experiments, *h*_max_ is equal to the time constant of the open-loop process. So, *h*_max_ determines the minimum sampling frequency. Figure 5 shows an example of the exchange of information among the control agents in the hybrid approach. The tuning parameters are *Kc*, *Ti*, *h*_sensor_, *h*_max_, *h*_controller_, and Δ_event_.

### 2.4. Software Architecture of the Experimental Framework

**Figure 6.**
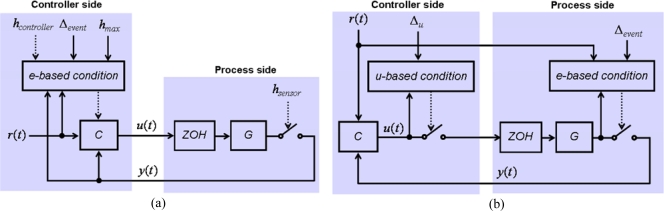
(a) Control scheme of the hybrid strategy. (b) Control scheme of the pure event-based control strategies

The control schemes developed to experiment with the hybrid and event-based criteria of Table 3 are illustrated in Figures 6.a and 6.b, respectively. The scheme is divided into two parts, the controller and the process side, both running in the same computer. The controller and process sides have been developed with Easy Java Simulations [30] and NI LabView, respectively. Both parts exchange data by a TCP/IP channel. The reason of using TCP/IP is to have ready a scheme to be able to perform further experiments with both sides running in different computers connected by Internet and so to analyze the impact of variable transmission delays in the performance of event-based strategies.

In both Figures 6, it can be appreciated that, regardless the selected error-based condition, the tuning parameters in the event-based control strategies are the PI controller parameters (*Kc* and *Ti*), Δ_event_, and Δ*_u_*.

**Figure 7.**
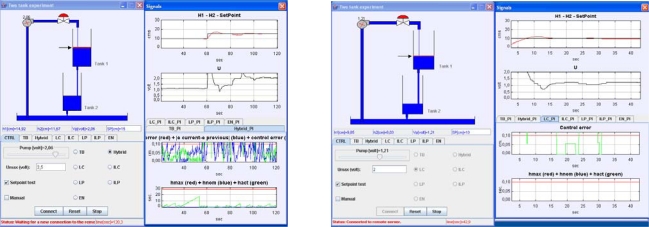
Client-side interfaces developed by Easy Java Simulations.

A view of the client-side interface developed in Easy Java Simulations is presented in Figure 7. Te graphical user interface is divided into two parts. The *main window* is located on the left side and it presents a graphical scheme of the plant. Below the scheme, there is a panel made of eight tabbed panels, one for each control strategy plus a panel with a common layout with options independent of the control algorithm applied (it is shown in Figures 7a,b). Some of these options are, for example, a text-field to change the set-point value (also it is possible to modify this value by dragging up and down the arrow located to the left of the upper tank), some non-editable text-fields with the evolution of some state variables, another text-field to fix the upper limit of the control action (that is, the maximum voltage applicable to the pump), a check-button to switch to manual control, a check-button to activate a time-varying set-point trajectory to check the control algorithms, or check-buttons to select the control algorithm. Each of the other seven tabbed panels presents a different layout with buttons, text-fields, and sliders that depend on the control algorithm that represent.

The *signals window* is placed on the right side and it is divided into two panels too. The upper panel is constituted by two signal scopes that present the common variables to any control algorithm: the first one shows the liquid level of the upper tank (trace in red) plus the set point value (trace in black); the second scope presents the evolution of the control action value. Below these scopes, there is a panel including six tabbed panels. All these panels hold signal scopes but each of them offers a different layout since each one displays the variables corresponding to a specific control algorithm. So, for example, Figure 7 presents the tabbed panel belonging to the hybrid strategy and the signals displayed are |*e*(*t*) − *e*(*t_k_*)|, *e*(*t*), Δ_event_, *h*_withouit_, *h*_max_, *h*_nom_.

## 3. Description of the Test-Bed and the Performance Criteria

### A. The Experimental Set-Up

**Figure 8.**
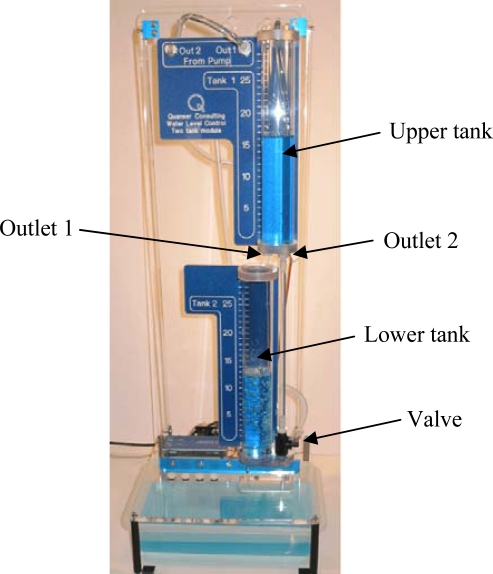
The two-tank system. In the experiments, just control level of the upper tank has been done. Disturbances are introduced into the system by the second outlet located in the upper tank bottom. This outlet is closed and open manually by a valve located near the bottom right of the lower tank.

As it was said before, the experiments have been carried on in a double-tank system where just level control of the upper tank is done (Figure 8). The double-tank is plugged into a computer with a DAQ with 14-bit ADC, ±5 V. analog inputs, 12 bit DAC, and ±5 V. analog outputs. By identification techniques and working just in the linear zone of the upper tank (from 10 to 15 cm.), we have obtained the following FOTD model:

$$P(s)=\frac{17.8}{14.2s+1}e^{-1.4s}$$

Applying tuning rules, we have obtained the following parameters for the time-based PI controller: *Kc* = 0.51, *Ti* = 4.6. The data obtained by the time-based strategy based on this controller will be the reference to compare and contrast the results of the hybrid and event-based strategies in next sections.

### B. Performance Criteria

To determine the performance of the hybrid and event-based control strategies, we have established two sets of criteria. The first set assesses the design quality regarding the reduction of the triggered number of asynchronous events (that is, the sendings from sensor to controller), the inter-events period, the events per second, the calculations of new control actions, and the sendings from the controller to the actuator. The second set calculates the quality of the system response. Also, a global index is calculated taking into account the efficiency of the sampling and the quality of the system response.

#### 1) Indexes on the sampling efficiency

- *Calls*: Measures the number of sendings from the sensor to the controller.
- *E_calls*: Ratio of the number of sensor-controller sendings between the hybrid and event-based approaches and the time-based.
- *Actions*: Number of invocations of the PI controller.
- *E_actions*: Ratio of the invocations of the PI controller between the hybrid and event-based approaches and the time-based.
- *Sendings*: Number of sendings of control actions from the controller to the actuator in the event-based approaches.
- *E_sendings*: Ratio of sendings of control actions between the hybrid and event-based approaches and the time-based.
- *T_average*: Average time between two consecutive events, that is, between two consecutive sendings from sensor to controller.
- *S_average*: Sendings sensor-controller per second.

#### 2) Indexes on the quality of the system response

- *IAE*: The integrated absolute error is defined as:
  $$\mathrm{IAE}=\int_{0}^{\infty }\left|e(t)\right|\mathrm{dt}$$
- *IAEP*: The integrated absolute difference between the system response of an hybrid and event-based strategies and the system response of the time-based:
  $$\mathrm{IAEP}=\int_{0}^{\infty }\left|y_{\mathrm{time\_based}}\;(t)-y_{\mathrm{event–based}}\;(t)\right|\mathrm{dt}$$
- *NE*: Another measure to compare the quality of the system response:
  $$\mathrm{NE}=\frac{\mathrm{IAEP}}{\mathrm{IAE}}$$
- *IAD*: The integrated absolute difference between the IAE of the time-based strategy and the IAE of the hybrid and event-based ones:
  $$\mathrm{IAD}=\int_{0}^{\infty }\left|{\mathrm{IAE}}_{\mathrm{event–based}}-{\mathrm{IAE}}_{\mathrm{time–based}}\right|\mathrm{dt}$$

#### 3) The global performance index

The GPI index [25] shows the compromise between the control performance and the sampling efficiency:

$$\mathrm{GPI}=W_{1}\cdot \mathrm{Calls}+W_{2}\cdot \mathrm{Actions}+W_{3}\cdot \mathrm{Sendings}+W_{4}\cdot \mathrm{NE}$$

A lower value of this index means that the control strategy presents a better global performance. In our experiments, the four weights have been set to one.

## 4. Results and Comments on the Experiments

We have designed two types of experiments to compare and contrast the performance of the event-based controllers in the two tasks any controller has to accomplish: a) the set-point following and b) the disturbance rejection.

In both cases, a first experiment was done with the time-based approach to get a basis for comparing and contrasting the other approaches. The values of the sampling periods were *h*_sensor_ = 0.1 ms and *h*_controller_ = 0.1 ms. After that, experiments with the hybrid-approach and the event-based ones were done for three values of Δ_event_. Regardless of the control approach, the duration of the experiment was always 60 s. In the experiments to test the performance of the controllers in the set-point following, we change the set-point value in the upper tank from 10 to 15 cm. Tables 4, 5, and 6 show the results for three different values of Δ_event_.

It is important to say the meaning and units of Δ_event_ depend on the error-based condition (see Table 3). So, Δ_event_ is expressed by centimeters in the hybrid approach, LC, and LP; by cm*sec in ILC and ILP; and by cm^2^*sec in EN.

Further it must be noticed that we present experiments with Δ*_u_* = 0 for all the control approaches. It means that every time a control action is computed, it is sent to the actuator; for this reason, the values of *Sendings* and *E_sendings* are equal to *Actions* and *E_actions*, respectively, and we have not included them in the tables for the sake of simplicity.

**Figure 9.**
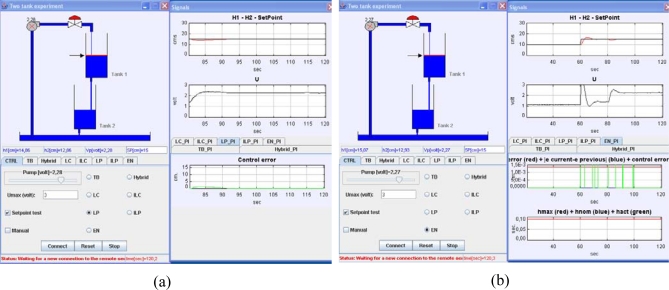
The disturbance is introduced at *t* = 80 s by opening the second outlet of the upper tank.

In the experiments to check the disturbance rejection ability, and regardless the control strategy, the length of the experiments (60 s.) and the values of the PI parameters were the same that in the group of experiments to assess the performance of the set-point tracking. The only difference was that data in Tables 7, 8, and 9 correspond to experiments where a disturbance was introduced in the output flow by the full opening of the second outlet located at the bottom of the upper tank (see Figure 8). In these experiments, the liquid level is set to 15 cm (*t* = 0 s) and in *t* = 20 s the outlet is open till the end of the experiment (*t* = 60 s) In our set-up, the opening of the second outlet produces an increase of the 25% in the output flow and the logical response of the controller must be an increase of the control action to reestablish the current set-point value, that is, 15 cm. As an example, Figures 9a and 9b show the system response and control action for Δ_event_ = 0.3 using the LP and EN approaches, respectively. In the signal scopes, it is possible to appreciate the increase of the controller action to reach the set-point value. Also, it must be noticed the good quality of the system responses in both cases since they present just a slight overshoot.

It produces an increase of the output flow in 13.618 cm^3^/s that corresponds to increment the output flow a 25%. **(a)** LP approach **(b)** EN approach. In both experiments, Δ_event_ = 0.3.

In the next paragraphs, we comment on the results of the experiments to assess the performance of the event-based and hybrid control approaches in the set-point following and the disturbance rejection tasks. Comments are divided into four sections: 4.1) on the sampling quality, 4.2) on the quality of the system response, 4.3) on the global performance index, and 4.4) on the manipulated variable.

In Section 4.1, we compare the number of sensor-controller sendings, the times that a new control action is calculated, the number of controller-actuator sendings, and the inter-event time. In Section 4.2, we check the quality of the system response by comparing the *IAE*, *IAEP*, and *NE* indexes. In Section 4.3, we assess the global performance of the approaches by combining the results of Sections 4.1 and 4.2. In Section 4.4, we comment the behaviour of the manipulated. It must be noticed that in Sections 4.1, 4.2, and 4.3, we comment the results regarding three different frameworks: A) set-point following, B) disturbance rejection, and C) increase of Δ_event_.

### 4.1. On the Sampling Efficiency

#### 4.1.A. Set-point following

- The time-based and the hybrid approaches present the same number of sensor-controller sendings (*Calls*). However, the invocation of the PI controller and the controller-actuator sendings (*Actions*) have been reduced a 92.6% in the hybrid case (Table 4).
- Regarding the event-based approaches, the sensor-controller sendings (*Calls*) have been considerably cut down.
- The LC approach presents the best value of *Calls* with a reduction of 92% with respect to the time-based and hybrid approaches. However, the *IAE* values are in general higher than in the other event-based approaches.
- We must underline the LP approach where a reduction of 87% is obtained with an *IAE* practically equal to the time-based approach (see Figure 12).

#### 4.1.B. Disturbance rejection task

- The invocations of the PI controller (*Calls*) and the controller-actuator sendings (*Actions*) are increased in every event-based algorithm. The highest increment corresponds to the EN algorithm: going from 210 to 336 invocations.
- The inter-event time (*T_average*) is cut down in all the approaches. The higher reduction corresponds to the EN algorithm that goes from 0.2857 s. to 0.178 s.
- The LC approach presents the smaller values of *Calls* and *Actions* but however the *IAE* index is not so good.
- The LP approach offers an effective quality in the sampling (*Calls*, *Actions*) with a good quality of the system response since the *IAE* is close to the time-based one.

#### 4.1.C. Increase of Δ_event_

- It has been possible to reduce an 88.5% the sensor-controller sendings (*Calls*) with the LP approach without a worsening of the system response.
- A reduction of events is observed in some event-based approaches, as it was expected. However, sometimes that situation does not happen (e.g., ILP and EN). In these approaches based on error integration, a higher Δ_event_ produces an increase of the events because the integrated error expression exceeds the threshold faster than in other event-based approaches. This is a situation where there is a clear difference between the use of an event-based approach for control purposes or for data transmissions. To increase Δ*_event_* should always mean a reduction of events, but does not happen in some cases.

### 4.2. On the Quality of the System Response

#### 4.2.A. Set-point following

- The worse response corresponds to the hybrid approach, followed by the LC.
- The ILC and ILP approaches show a similar behaviour to the time-based approach.
- The EN approach presents some oscillations in the response and it produces a high *IAEP*.

#### 4.2.B. Disturbance rejection

- In all the event-based approaches, the *IAE* index is increased because of the disturbance. The Hybrid approach presents the higher increase since it goes from 2.59 × 10^4^ to 6.79 × 10^4^.
- The *IAEP* index is also raised in every algorithm. It demonstrates a worsening of the system response when comparing with the time-based approach. This deterioration is consequence of the transitory in the system response when the disturbance is introduced. The *NE* index is increased in all the algorithms with the exception of the LP. In this case, the *NE* value goes from 0.1902 to 0.1829. It is an indicator of the good quality of the response of this algorithm.
- The *IAD* index has significant grown in the Hybrid and LC approaches. It shows an increment of the difference in the error of the system responses between the event-based and the time-based approaches. The most significant increase corresponds to the Hybrid approach; in the other approaches it is appreciated a moderate decrement of this index value.
- The ILP approach shows a very good quality of the system response (*IAE*, *IAEP*) when comparing with the other event-based approaches. The system response of the ILP is very close to the time-based counterpart, but with a reduction of *Calls* and *Actions* going from 600 to 283.
- In all the event-based approaches, the disturbance is rejected with a smooth overshooting. It demonstrates the ability of the event-based approaches to reject constant disturbances.

#### 4.2.C. Increase of Δ_event_

**Figure 10.**
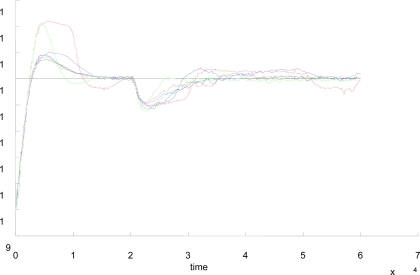
System responses of the time-based (black), hybrid (red), LC (blue), ILC (green), LP (dashed red), ILP (dashed blue), EN (dashed green) with disturbance. Δ_event_ = 0.2.

**Figure 11.**
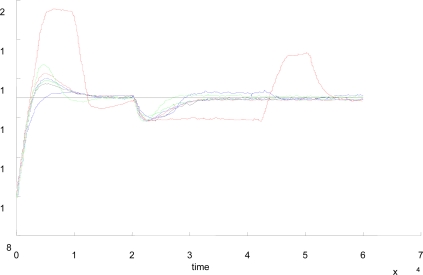
System responses of the time-based (black), hybrid (red), LC (blue), ILC (green), LP (dashed red), ILP (dashed blue), EN (dashed green) with disturbance. Δ_event_ = 0.3.

- In general, the quality of the system responses gets worse.
- The ILP and ILC present the best results in the experiments to asses the set-point following task (Tables 4, 5, and 6)
- En general, the increase does not produce a relevant worsening since, for example, the LP or the EN approaches get very similar responses to the experiments with Δ_event_ = 0.1 and 0.2 (Tables 8 and 9, Figures 10 and 11).

### 4.3. On the Global Performance Index (GPI)

#### 4.3.A. Set-point following

- The approach with the best global behaviour is the LC. It is consequence of the significant sendings (*Calls*) reduction without a relevant deterioration of the system response.
- The event-based approaches based on the integral (ILC, ILP, NE) present a high *GPI*. It is due to the number of sendings (*Calls*) in spite of the good quality of the system responses (*IAE*). It is consequence that the sampling efficiency has a bigger weight in the *GPI* expression than the quality response factor.

#### 4.3.B. Disturbance rejection

- The *GPI* index has grown in all the approaches because of the increment of the *Calls* and *Actions*.
- The lowest *GPI* corresponds to the LC approach as a consequence of the reduced values of *Calls*, *Actions*, and *Sendings* and good values of *IAE*, *IAEP*, and *NE*. It means a better quality of the system response when comparing with the other approaches.
- The LP approach presents a good *GPI* value, a reduced number of *Calls* and *Actions*, and a quality response on the average of the other event-based approaches.

#### 4.3.C. Increase of Δ_event_

- In the set-point following experiments, the best approach is the LC. It shows the lowest number of calls (*Calls*) and the response quality is not very bad, especially with Δ_event_ = 0.3.
- It is clear that Δ_event_ must be tuned regarding the event-based approach. So, the same value of Δ_event_ in ILP/ILC produces a higher number of sending than in LP/LC but, however, the response quality is better. A higher Δ_event_ in ILP/ILC should reduce the *GPI* index as consequence of the reduction of the sendings (*Calls*).
- In the experiments in presence of disturbances (Tables 8 and 9), the best approach is the LC. However, the *IAE* value is not the best.
- It must be noticed that the LP approach presents a good *GPI* in both tables and also the values of the *IAE* and *T_average* are good and, in some experiments, even better that the LC ones.
- It must be noticed that the LP approach presents a good *GPI* in both tables and also the values of the *IAE* and *T_average* are good and, in some experiments, even better that the LC ones.

### 4.4. On the Manipulated Variable

**Figure 12.**
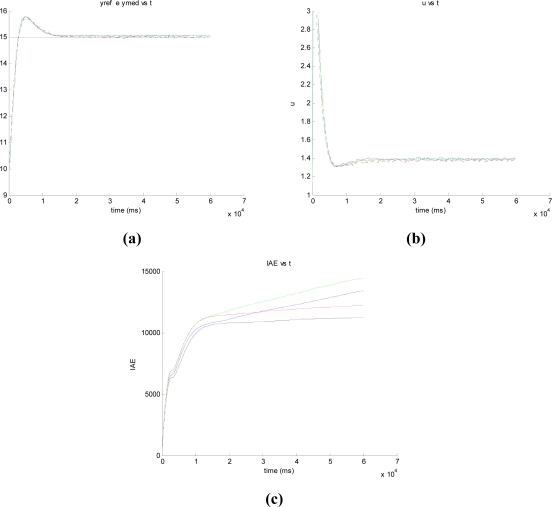
**(a)** System response, **(b)** manipulated variable, and **(c)** IAE of the time-based (black) and LP approaches with Δ_event_ = 0.1 (red), 0.2 (blue), and 0.3 (green).

**Figure 13.**
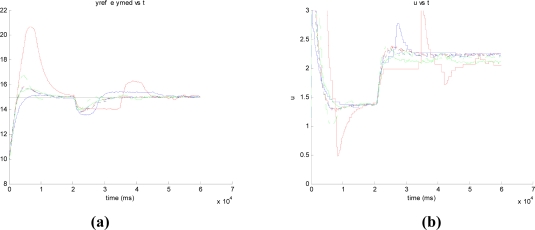
**(a)** System responses and **(b)** control action of the time-based (black), hybrid (red), LC (blue), ILC (green), LP (dashed red), ILP (dashed blue), EN (dashed green) with disturbance. Δ_event_ = 0.1.

Figure 12b presents the evolution of the manipulated variable using the time-based and the LP approaches with different Δ_event_. These values have been obtained with the experiment designed to assess the performance of the set-point tracking. When the set-point value is changed from 10 to 15 cm and the steady state of the process is reached, the manipulated variable gets a value of 1.4 V. Initially, the control action reaches its upper limit (3 V), falling quickly until reaching its stable final value. It must be noticed that the evolution of the manipulated variable is very similar in both approaches, and there is not a clear difference between both system responses. In the experiments with disturbance (Figure 13), the manipulated variable in the LP approach goes from 1.4 to 2.3 V in the steady state. Such change is for increasing the input flow in order to compensate the increase of the output flow due to the disturbance.

Before reaching the steady state, the hybrid approach produces a sharp change of the manipulated variable that reaches its upper limit with some oscillations. The LC approach produces a jump of the manipulated variable, reaching 2.75 V; this increase is quickly cut down, reaching a value nearly constant, slightly higher than in the other event-based approaches.

Regarding the other event-based approaches, the evolution of the manipulated variables is almost similar to the time-based. However, it is important to notice that the control action in the EN approach offers lower values than in the time-based controller.

## 5. Conclusions and Further Work

We have presented an experimental study of some sampling and control strategies based on events. Many experiments have been carried out to compare and contrast five pure event-based control approaches with a classical time-based approach and a hybrid one. These experiments have been divided into two classes, corresponding to the two main tasks that any controller must carry out: (1) experiments to evaluate the set-point following task and (2) experiments to assess the disturbance rejection task.

Also, a new event-based approach, named ILP, has been introduced with acceptable results. It is important to notice that after a detailed study of the tables and results, the LP approach is not the best option according to the global performance index (*GPI*) in the set-point following task, but, nevertheless, it produces very balanced results regardless of the value of Δ_event_ in both control tasks. If we concentrate our attention in the sensor-controller sendings (*Calls*), the quality of the system response (*IAE*), and the average time between two consecutive events (*T_average*), the LP approach is the choice. From the results of this experimental study, we can state that the best options are the two approaches with no integration (LC and LP) but it requires the introduction of time-triggered conditions to avoid the sticking.

We can assert that event-based approaches are convenient control strategies when the key design constraint is the reduction of the exchange of information between the control agents (sensors, controllers, actuators) or the reduction of the computational load. As penalty we would have a light worsening of the system response in some cases.

However, there are open questions to be solved in future research. First, the absence of design procedures to tune the different parameters involved in an event-based control strategy, especially Δ_event_ and its relationship with the other parameters (*Kc* and *Ti* in a PI controller). There are recommendations but they are result of experimental practices, and much theory is required. Also, a simplest generalized performance index *GPI* should be researched since the current expression is very user-dependant and it produces misleading conclusions as, for example, happens with the LP approach in this work. A new *GPI* should take into account the time between events since it is a fundamental argument when these approaches are meant to be used in battery-powered devices with wireless communication.

At the time being, part of our research on events is focused on deriving expressions of minimum boundaries of Δ_event_ for the event-based approaches, in establishing equivalence relationships among the event-based strategies, and in defining when one event-based approach is more convenient than others regarding the features and communication channels of the control agents involved. Also, to study the behaviour of the event-based approaches in the frequency domain we are looking for non-linearities that produce similar results to the sampling based on error-based conditions. By obtaining the corresponding descriptive functions, we will be able to study the behaviour in the frequency domain and the existence of oscillations and limit cycles.

## Figures and Tables

**Table 1.** Control agents, events, and actions.

**AGENT**	**EVENT**	**ACTION**
Sensor	Fulfill error-based criterion	Send *y*
Sensor	Time-out	Send *y*
Actuator	New *u*	Apply *u*
Controller	New reference (input side)	Calculate *u*
Controller	New *y* (input side)	Calculate *u*
Controller	Fulfill u-based criterion (output side)	Send *u*

**Table 2.** Nature of the control agents in the three control approaches.

**APPROACH (total strategies)**	**SENSOR**	**CONTROLLER**	**ACTUATOR**
Time-driven (1)	Time-driven	Time-driven	Event-driven
Hybrid (1)	Time-driven	Time/event-driven	Event-driven
Event-based (5)	Event-driven	Event-driven	Event-driven

**Table 3.** Error-based conditions of the sampling strategy applied to the sensor.

**Error-based condition**	**Label**	**It becomes true when:**
|*e*(*t*) − *e*(*t_k_*)| ≥ Δ_*event*_	LC	The difference between the current error and the value of the error the last time that condition was true is greater than Δ_event_.
$\int_{k}^{k+1}\left|e(t)-e\left(t_{k}\right)\right|\mathrm{dt}\geq \Delta_{\mathrm{event}}$	ILC	The value of the IAE from the last time that the condition was true is greater than Δ_event_.
|*ê*(*t*) − *e*(*t*)| ≥ Δ_*event*_	LP	The difference between a prediction of the error and its current value is greater than Δ_event_.
$\int_{k}^{k+1}\left|\hat{e}(t)-e(t)\right|\mathrm{dt}\geq \Delta_{\mathrm{event}}$	ILP	The integral of the difference between the prediction and the error is greater than Δ_event_.
$\int_{k}^{k+1}{\left[e(t)-e\left(t_{k}\right)\right]}^{2}\mathrm{dt}\geq \Delta_{\mathrm{event}}$	EN	The energy of the difference between the current error and the error last time condition was true is greater than Δ_event_.

**Table 4.** Performance indexes of the hybrid and event-based approaches for Δ*_event_* = 0.1. Figures in bold represent the best value of every performance index in the hybrid and event-based approaches.

**Index**	**Time-based**	**Hybrid**	**LC**	**ILC**	**LP**	**ILP**	**EN**

*Calls*	600	600	**47**	257	77	264	210
*E_calls*	1	1	0.078	0.4283	0.1283	0.44	0.35
*Actions*	600	44	**47**	257	77	264	210
*E_actions*	1	0.073	0.078	0.4283	0.1283	0.44	0.35
*T_average*	0.1	0.1	**1.276**	0.233	0.78	0.223	0.286
*S_average*	10	10	0.783	4.283	1.283	4.4	3.5
*IAE*	1.12 × 10^4^	2.59 × 10^4^	1.8 × 10^4^	**1.18 × 10**^4^	1.25 × 10^4^	1.22 × 10^4^	1.41 × 10^4^
*IAEP*	0	**1.59 × 10^3^**	1.02 × 10^4^	1.87 × 10**^3^**	2.37 × 10^3^	2.01 × 10^3^	6.85 × 10^3^
*NE*		0.613	0.5672	**0.1585**	0.1902	0.1641	0.4861
*IAD*	0	**6.21 × 10**^4^	2.23 × 10^8^	2.57 × 10^7^	5.65*10^7^	4.76 × 10^7^	1.65 × 10^8^
*GPI*		1244.6	**141.567**	771.158	231.19	792.16	630.486

**Table 5.** Performance indexes of the hybrid and event-based approaches for Δ_event_ = 0.2. Figures in bold represent the best value of every performance index in the hybrid and event-based approaches.

**Index**	**Time-based**	**Hybrid**	**LC**	**ILC**	**LP**	**ILP**	**EN**

*Calls*	600	600	**48**	249	68	279	228
*E_calls*	1	1	0.08	0.4150	0.1133	0.4650	0.38
*Actions*	600	73	**48**	249	68	279	228
*E_actions*	1	0.1217	0.08	0.4150	0.1133	0.4650	0.38
*T_average*	0.1	0.1	**1.25**	0.24	0.8823	0.215	0.2631
*S_average*	10	10	0.8	4.15	1.13	4.65	3.8
*IAE*	1.12 × 10^4^	6.33 × 10^4^	2.09 × 10^4^	**1.32 × 10^4^**	1.41 × 10^4^	1.34 × 10^4^	1.55 × 10^4^
*IAEP*	0	5.37 × 10^3^	1.16 × 10^4^	**3.25 × 10^3^**	4.04 × 10^3^	3.53 × 10^3^	7.93 × 10^3^
*NE*		0.8484	0.5548	**0.2465**	0.2857	0.2628	0.5128
*IAD*	0	1.91 × 10^8^	3.32 × 10^8^	**5.43 × 10^7^**	8.1 × 10^7^	5.54 × 10^7^	1.9 × 10^8^
*GPI*		1273.8	**144.55**	747.246	204.286	837.26	684.513

**Table 6.** Performance indexes of the hybrid and event-based approaches for Δ_event_ = 0.3. Figures in bold represent the best value of every performance index in the hybrid and event-based approaches.

**Index**	**Time-based**	**Hybrid**	**LC**	**ILC**	**LP**	**ILP**	**EN**

*Calls*	600	600	**42**	253	67	282	252
*E_calls*	1	1	0.07	0.4217	0.1117	0.47	0.42
*Actions*	600	93	**42**	253	67	282	252
*E_actions*	1	0.155	0.07	0.4217	0.1117	0.47	0.42
*T_average*	0.1	0.1	**1.4285**	0.2371	0.8955	0.2217	0.238
*S_average*	10	10	0.7	4.216	1.116	4.7	4.2
*IAE*	1.12*10^4^	2.19 × 10^4^	1.88 × 10^4^	**1.42 × 10^4^**	1.57 × 10^4^	1.45 × 10^4^	1.51 × 10^4^
*IAEP*	0	1.15 × 10^4^	8.32 × 10^3^	4.26 × 10^3^	5.69 × 10^3^	**3.97 × 10^3^**	7.91 × 10^3^
*NE*		0.5247	0.4408	0.3003	0.3613	**0.2746**	0.5244
*IAD*	0	5.1 × 10^8^	2.91 × 10^8^	**7.67 × 10^7^**	1.5 × 10^8^	9.95 × 10^7^	2.093 × 10^8^
*GPI*		1293.5	**126.44**	759.3003	201.36	846.27	756.52

**Table 7.** Performance of the hybrid and event-based approaches for Δ_event_ = 0.1 with disturbance. Figures in bold represent the best value of every performance index in the hybrid and event-based approaches.

**Index**	**Time-based**	**Hybrid**	**LC**	**ILC**	**LP**	**ILP**	**EN**

*Calls*	600	600	**54**	274	93	283	336
*E_calls*	1	1	0.09	0.457	0.155	0.472	0.56
*Actions*	600	98	54	262	93	283	336
*E_actions*	1	0.163	0.09	0.437	0.155	0.4717	0.56
*T_average*	0.1	0.1	**1.11**	0.218	0.645	0.212	0.178
*S_average*	10	10	0.9	4.56	1.55	4.716	5.6
*IAE*	1.9*10^4^	6.79 × 10^4^	2.75 × 10^4^	1.92 × 10^4^	1.96 × 10^4^	1.90 × 10^4^	**1.99 × 10^4^**
*IAEP*	0	5.15 × 10^4^	1.89 × 10^4^	5.08 × 10^3^	3.59 × 10^3^	**3.36 × 10^3^**	1.22 × 10^4^
*NE*		0.758	0.69	0.2641	0.1829	**0.1766**	0.6608
*IAD*	0	2.11 × 10^9^	2.14 × 10^9^	2.56 × 10^7^	4.33 × 10^7^	**1.55 × 10^7^**	9.30 × 10^7^
*GPI*		1248.8	**168.7**	822.3	279.2	849.18	1008.7

**Table 8.** Performance of the hybrid and event-based approaches for Δ_event_ = 0.2 with disturbance. Figures in bold represent the best value of every performance index in the hybrid and event-based approaches.

**Index**	**Time-based**	**Hybrid**	**LC**	**ILC**	**LP**	**ILP**	**EN**

*Calls*	600	600	**64**	281	90	291	363
*E_calls*	1	1	0.107	0.468	0.15	0.485	0.605
*Actions*	600	69	**64**	281	90	291	363
*E_actions*	1	0.115	0.1067	0.468	0.15	0.485	0.605
*T_average*	0.1	0.1	**0.937**	0.213	0.66	0.206	0.165
*S_average*	10	10	1.066	4.683	1.5	4.85	6.05
*IAE*	1.9 × 10^4^	4.02 × 10^4^	2.64 × 10^4^	2.04 × 10^4^	**1.91 × 10^4^**	2.03 × 10^4^	1.99 × 10^4^
*IAEP*	0	2.47 × 10^4^	1.12 × 10^4^	**4.02 × 10^3^**	6.17 × 10^3^	5.02 × 10^3^	1.35 × 10^3^
*NE*		0.612	0.423	**0.197**	0.323	0.247	0.679
*IAD*	0	8.04 × 10^8^	2.24 × 10^8^	4.52 × 10^7^	**3.19 × 10^7^**	5.48 × 10^7^	1.15 × 10^8^
*GPI*		1269.6	**192.4**	843.2	270.32	873.25	1089.7

**Table 9.** Performance of the hybrid and event-based approaches for Δ_event_ = 0.3 with disturbance. Figures in bold represent the best value of every performance index in the hybrid and event-based approaches.

**Index**	**Time-based**	**Hybrid**	**LC**	**ILC**	**LP**	**ILP**	**EN**

*Calls*	600	600	**56**	277	95	284	335
*E_calls*	1	1	0.093	0.462	0.158	0.473	0.558
*Actions*	600	93	**56**	277	95	284	335
*E_actions*	1	0.155	0.093	0.462	0.158	0.473	0.558
*T_average*	0.1	0.1	**0.9524**	0.2166	0.6315	0.2112	0.179
*S_average*	10	10	1.05	4.616	1.583	4.733	5.583
*IAE*	1.9 × 10^4^	8.24 × 10^4^	2.16 × 10^4^	**2.05 × 10^4^**	2.34 × 10^4^	2.23 × 10^4^	2.12 × 10^4^
*IAEP*	0	6.50 × 10^4^	1.22 × 10^4^	6.21 × 10^3^	6.81 × 10^3^	**5.73 × 10^3^**	1.39 × 10^4^
*NE*		0.789	0.565	0.303	0.290	**0.257**	0.653
*IAD*	0	2.21 × 10^9^	**6.01 × 10^7^**	6.18 × 10^7^	1.63 × 10^8^	1.34 × 10^8^	1.29 × 10^8^
*GPI*		1293.8	**168.56**	831.3	285.29	852.25	1005.7
